# Apert Syndrome: An Insight Into Dentofacial Features

**DOI:** 10.7759/cureus.17735

**Published:** 2021-09-05

**Authors:** Bijimole Jose, Tharian B Emmatty, John Joseph Methippara, Kavita Kumar, Nidhi Mary Thampi

**Affiliations:** 1 Pediatric and Preventive Dentistry, Annoor Dental College & Hospital, Muvattupuzha, IND; 2 Pediatric and Preventive Dentistry, Annoor Dental college & Hospital, Muvattupuzha, IND; 3 Pediatric Dentistry, Innocent Smiles Dental Clinic, Ernakulam, IND

**Keywords:** aperts syndrome, craniosynostosis, syndactyly, dentofacial features, midface hypoplasia, pseudo cleft palate

## Abstract

Apert syndrome is a developmental malformation characterised by craniosynostosis (premature fusion of cranial sutures), midface hypoplasia, and syndactyly of hands and feet. Early synostosis of the coronal suture, cranial base, as well as agenesis of the sagittal suture, result in characteristic appearance and dental features like maxillary transverse and sagittal hypoplasia with concomitant dental crowding, a pseudo-cleft palate, and skeletal and dental anterior open bite. In this report, we discuss a case of Apert syndrome, with special emphasis on craniofacial characteristics, a multidisciplinary approach to its treatment, and the dentist’s role in management.

## Introduction

Apert syndrome is a rare congenital type I acrocephalosyndactyly syndrome affecting the first branchial arch. It is characterised by craniosynostosis, severe syndactyly of the hands and feet, symphalangism, and dysmorphic facial features [1]. The incidence of Apert syndrome is about 15 per 1,000,000 live births [2]. Although it was first reported by Wheaton [3] in 1894, French pediatrician Eugene Apert published a series of nine cases that exhibited a triad of craniosynostosis, syndactyly, and maxillary hypoplasia in 1906, and ever since then, his name has been associated with acrocephalosyndactyly [4].

Apert syndrome can be inherited in an autosomal dominant fashion; however, most cases are sporadic and associated with advanced paternal age [5]. Its etiology can also be attributed to two specific mutations of a gene located on chromosome 10q26, encoding fibroblast growth factor receptor 2 (FGFR2). More severe craniofacial anomalies are associated with S252W mutation occurring in 67% of patients and severe syndactyly with P253R mutation [6]. These mutations affect the region linking the immunoglobulin-like domains II and III of FGFR2 and result in increased affinity and altered specificity of ligand binding [7]. This in turn leads to deregulation of cell migration, proliferation, and differentiation and ultimately to premature osteogenesis and skeletal abnormalities that characterise the syndrome.

The coronal synostosis and the sagittal and metopic suture agenesis coupled with early synostosis of the cranial base results in acrocephaly, brachycephaly, flat occiput and high prominent forehead, hypoplastic midface, and a vertically accentuated craniofacial complex [8]. Eyes exhibit downward-slanting palpebral fissures, hypertelorism, shallow orbit, proptosis, and exophthalmos. The nose has a marked flat nasal bridge. Additionally, the maxilla is hypoplastic and retro-positioned. The palate is high-arched and narrow due to poor aeration in maxillary antra [9]. There are bulbous lateral palatal swellings, which make the central furrow of the palate very prominent and difficult to cleanse. Pseudo-cleft palate along with an anteriorly tipped palatal plane is very common [10]. The maxillary arch is V-shaped and slants down posteriorly, resulting in an anterior open bite. Severe crowding of developing teeth within the alveolus, delayed eruption, impactions, thick gingiva, and, sometimes, supernumerary or congenitally missing teeth are seen [11]. In the mandible, these findings are less pronounced. Skull radiographic findings like copper-beaten/beaten-metal appearance are seen in craniosynostosis due to the prominence of convolutional marking. The lips are characterised by the crossbow shape of the upper lip or the trapezoidal shape of both lips.

Cervical spine fusion occurs in up to 71% of patients with Apert syndrome and most often involves the fifth and sixth vertebrae [12]. Individuals become mouth breathers of necessity due to reduced airway patency with resultant anterior open bite. Syndactyly involves partial or complete fusion of second, third, and fourth digits [13]. Intelligence varies from normal to subnormal. Papilledema optic atrophy may be associated and hyperhidrosis is commonly seen. Cardiovascular manifestations like atrial septal defect (ASD), ventricular septal defect (VSD), patent ductus artery (PDA), and pulmonary stenosis may be present as well [14].

## Case presentation

A 12-year-old female patient presented to the Department of Paediatric and Preventive Dentistry with a complaint of pain in the lower-right back region for a few days. The pain was of sudden onset and the lingering type and increased in intensity at night. The patient presented with unusual craniofacial and dental features, which prompted a further detailed evaluation of the case. On questioning, the patient was revealed to be a known case of Apert syndrome. She was the first child born to non-consanguineous parents. Her mother's pregnancy and labour had been uneventful, and there had been no history of drug use during the entire term of pregnancy. The patient had two younger siblings who were normal. There was no family history of similar complaints, other congenital abnormalities, or advanced paternal age. She had undergone craniotomy at the age of one, as recommended by a neurologist, to advance her forehead and face.

**Figure 1:**
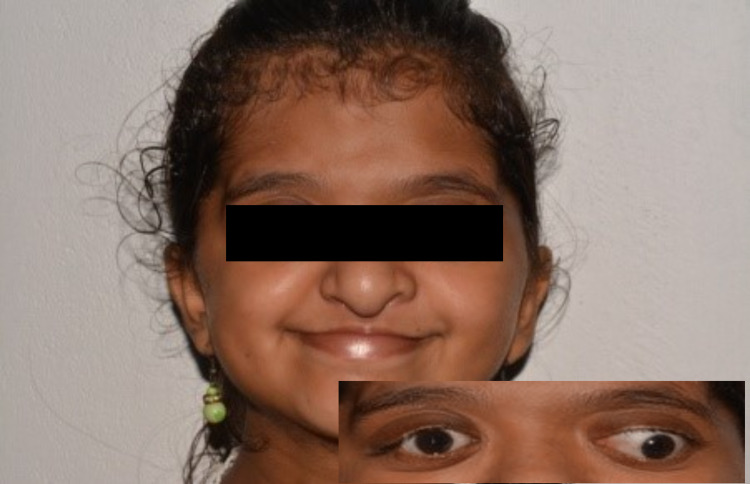
Extraoral features in the patient The image in the inset shows proptosis and hypertelorism

**Figure 2:**
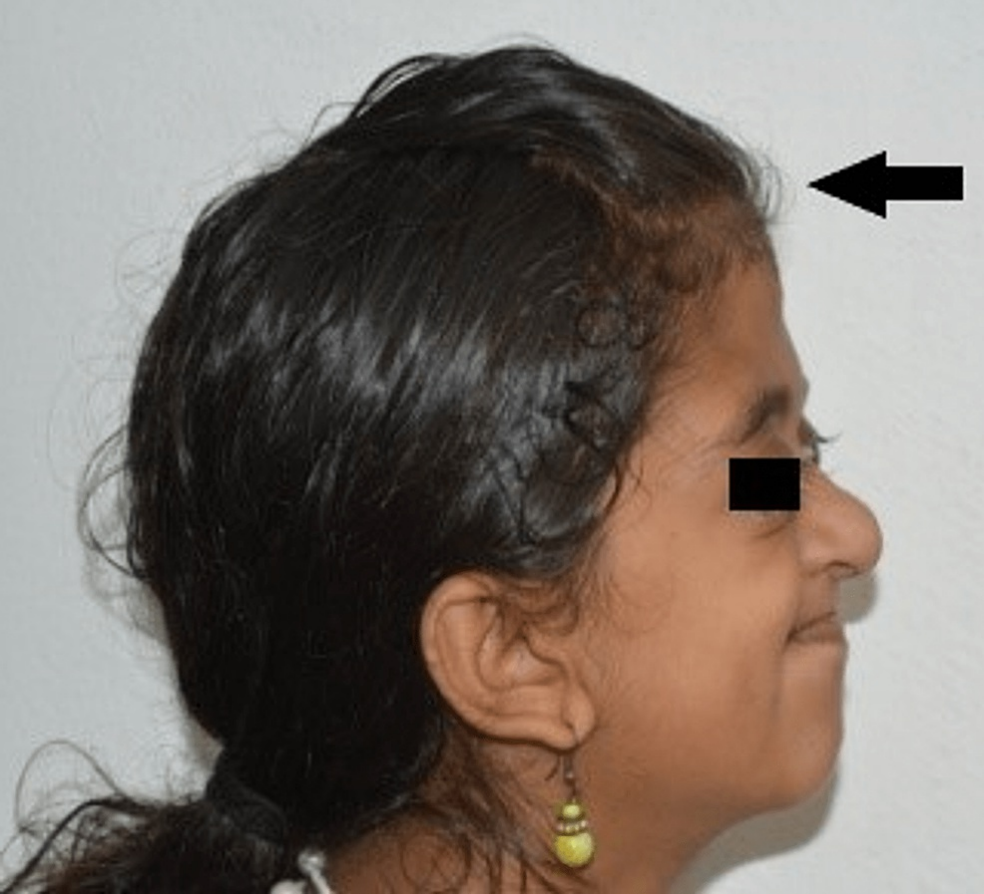
Profile view of the patient Arrow shows acrocephaly

Extraoral examination revealed that she had abnormal facies evidenced by acrocephaly, brachycephaly, a flat occiput, and a high, prominent forehead. Hypertelorism, ocular proptosis, downward-slanting palpebral fissures, depressed nasal bridge, and low set ears were also observed (Figures 1, 2).

**Figure 3:**
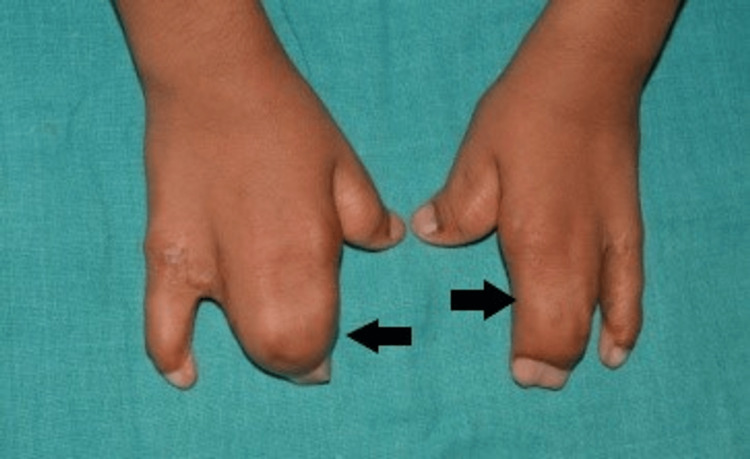
Syndactyly of the hands (arrows) - image 1

**Figure 4:**
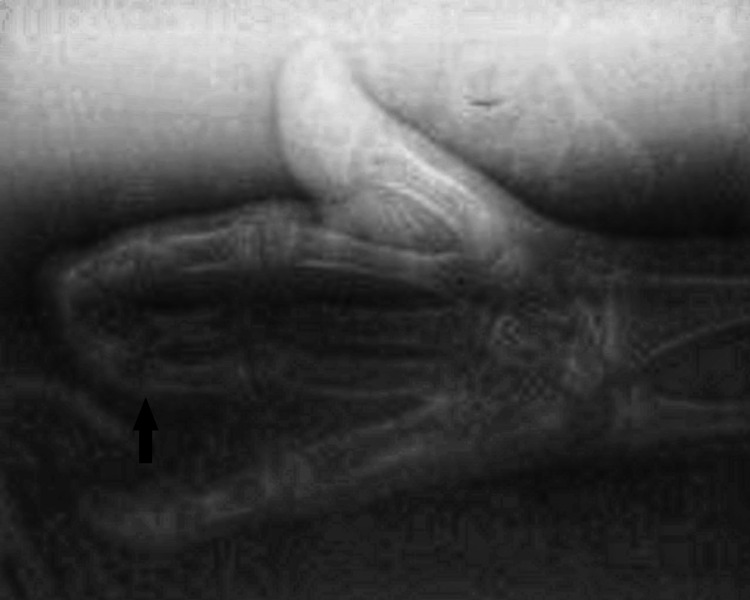
Syndactyly of the hands (arrow) - image 2

**Figure 5:**
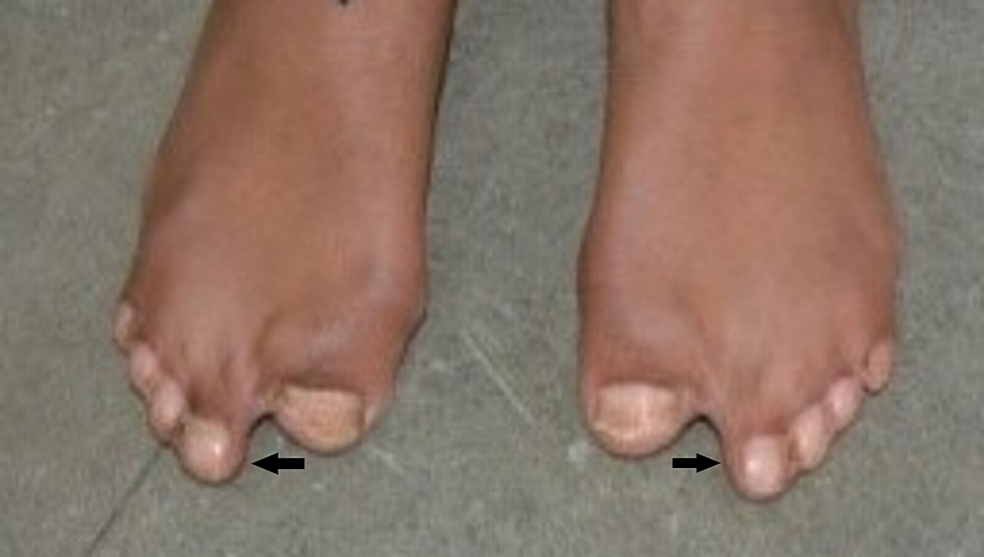
Syndactyly of the feet (arrows)

Syndactyly involving the hands and feet, causing a spoon-like deformity, was also present (Figures 3, 4, 5).

**Figure 6:**
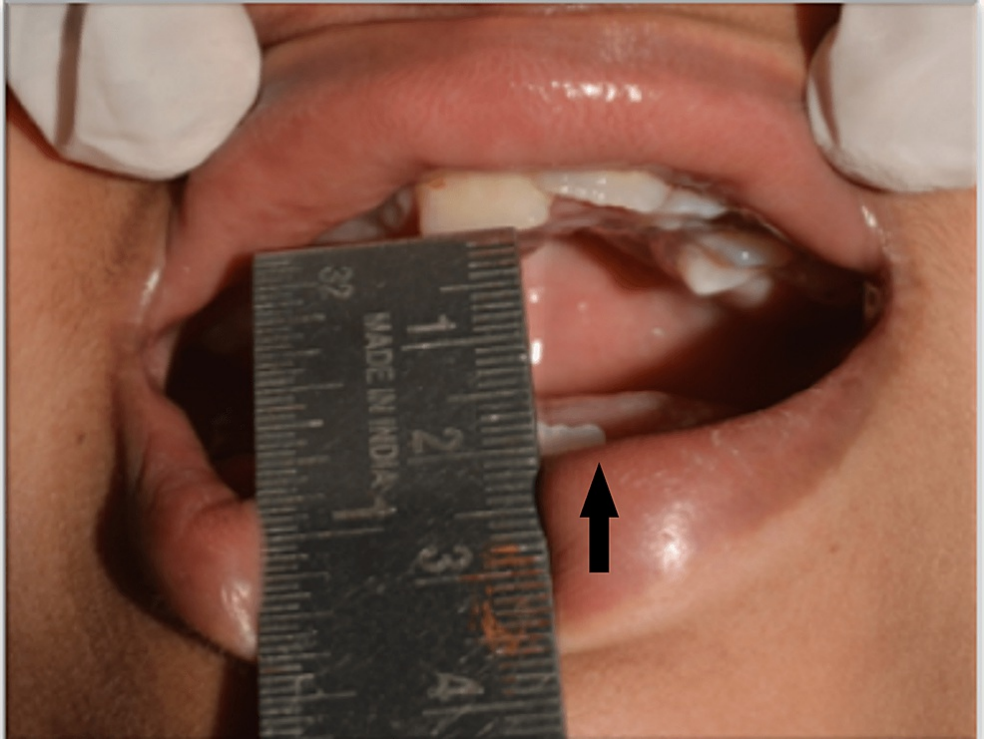
Decreased mouth opening (arrow)

On intraoral examination, all teeth were observed to be erupted into the oral cavity except for the maxillary right canine, and second and third molars. Micrognathia and decreased mouth opening were also observed (Figure 6).

**Figure 7:**
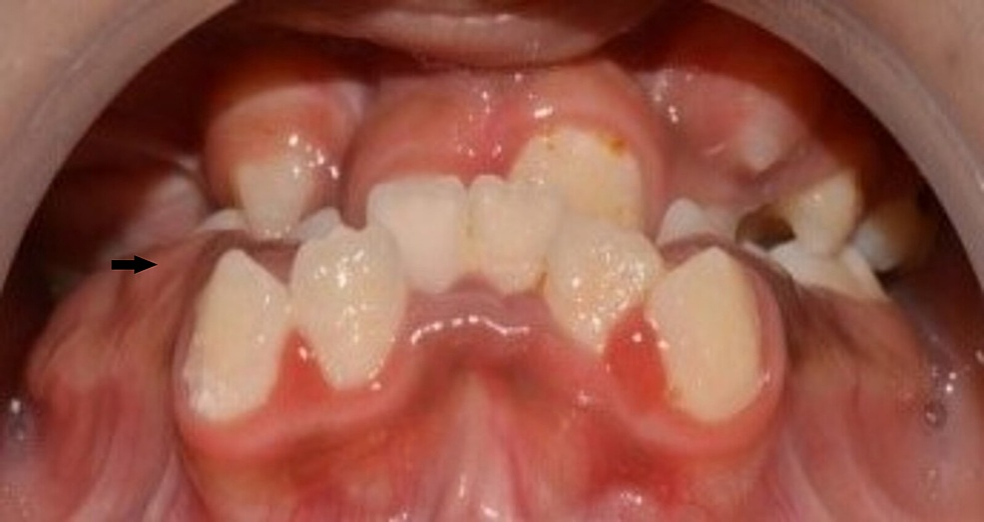
Occlusion (arrow)

**Figure 8:**
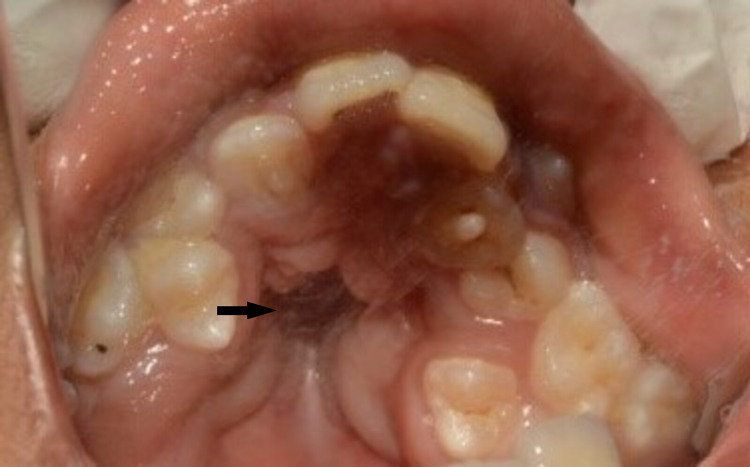
Maxilla occlusal view The arrow shows the pseudo-cleft

**Figure 9:**
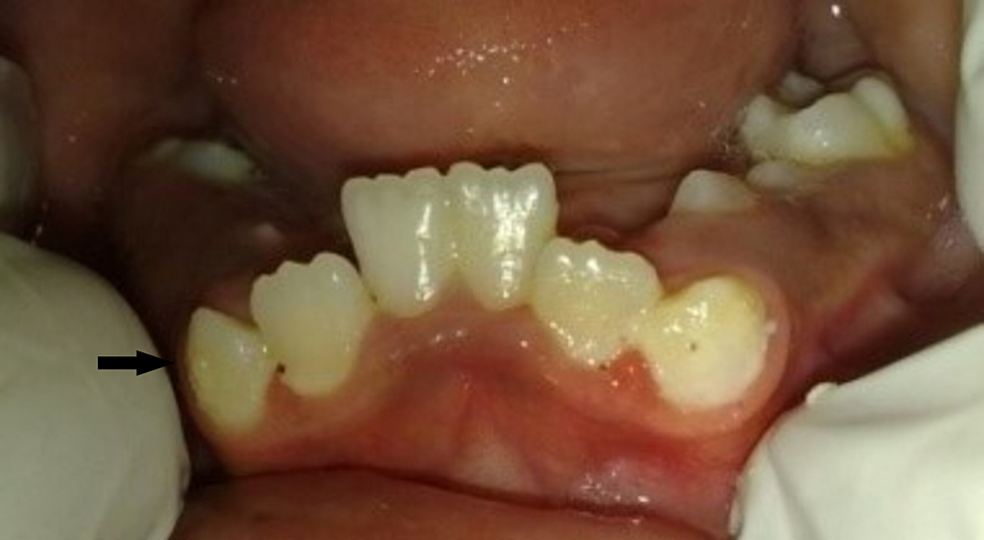
Mandibular arch (arrow)

Mandibular permanent right first molar was diagnosed as chronic apical periodontitis and left first molar dentinal caries. Typical dental and skeletal findings of Apert syndrome were observed upon intraoral examination. The palate was high-arched, with a pseudo-cleft in the posterior third. There was severe maxillary and mandibular teeth crowding. The patient had a dolichocephalic facial pattern with a class III molar and canine relation, an anterior-posterior crossbite, a deep bite, delayed eruption, and poor oral hygiene (Figures 7, 8, 9).

**Figure 10:**
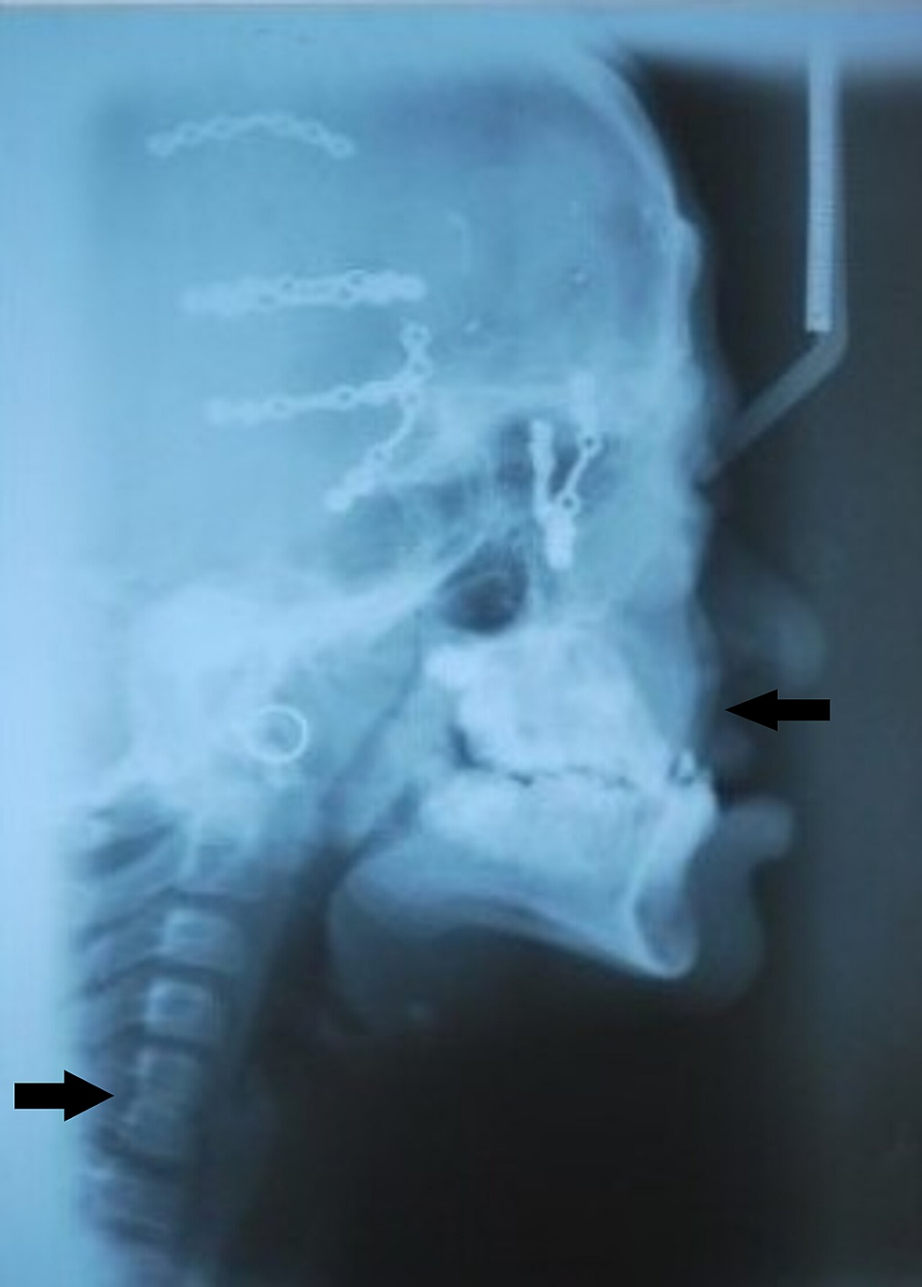
Lateral cephalometric finding Arrows show midface hypoplasia and cervical spine fusion

Upon lateral cephalogram, midface hypoplasia, upper airway constriction, and cervical spine fusion involving the fifth and sixth vertebrae were noted (Figure 10).

**Figure 11:**
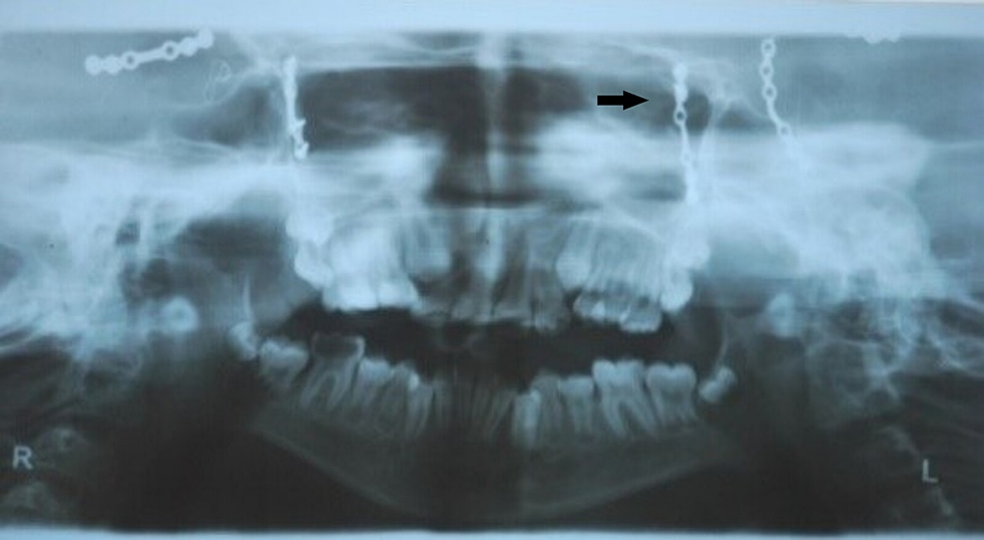
OPG Panoramic radiograph shows crowding and surgical plates OPG: orthopantomogram

On the panoramic radiograph, crowding and craniotomy were noted (Figure 11).

**Figure 12:**
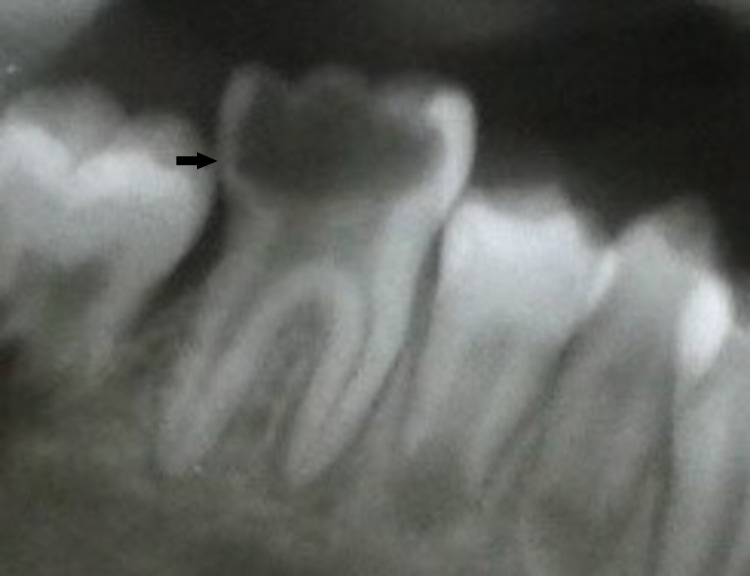
Preoperative X-ray 46: coronal radiolucency extending to the pulp chamber and periapical radiolucency present in mesial and distal roots

**Figure 13:**
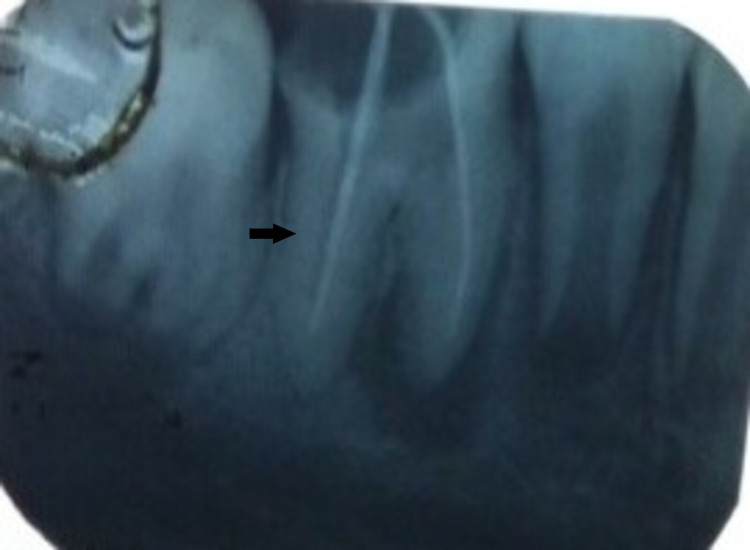
Working length X-ray

**Figure 14:**
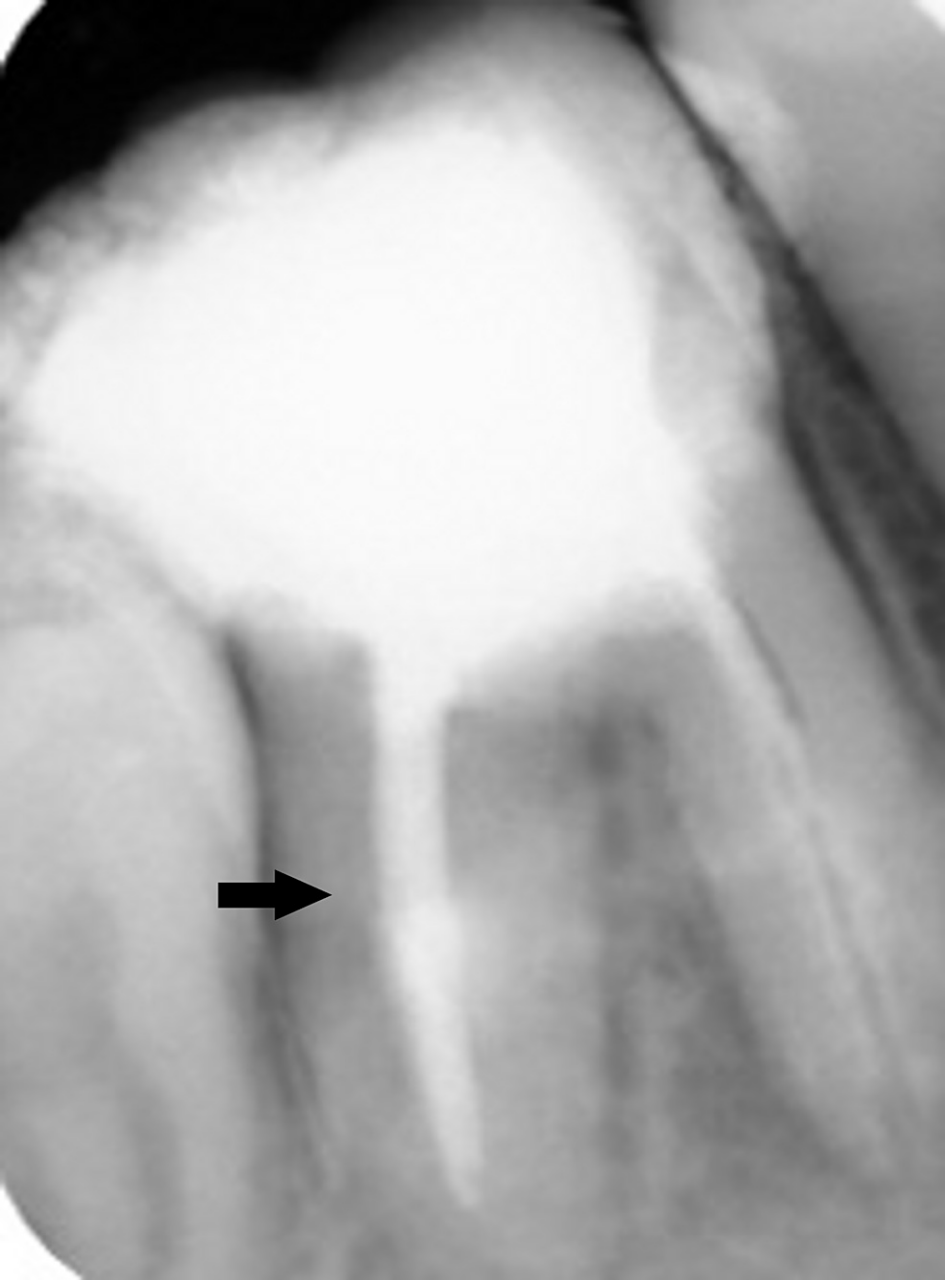
Obturation

Prior to the treatment, informed consent was obtained. Patient and parent education and counselling and motivation were given. To improve oral hygiene, oral prophylaxis was performed. An electric toothbrush was advised for toothbrushing. The preventive programme was started with a topical fluoride application [1.23% acidulated phosphate fluoride (APF)]. Carious 36 was restored with resin-modified glass ionomer cement (RMGIC). Root canal treatment was performed on 46, followed by the installation of a stainless steel crown (Figures 12, 13, 14).

**Figure 15:**
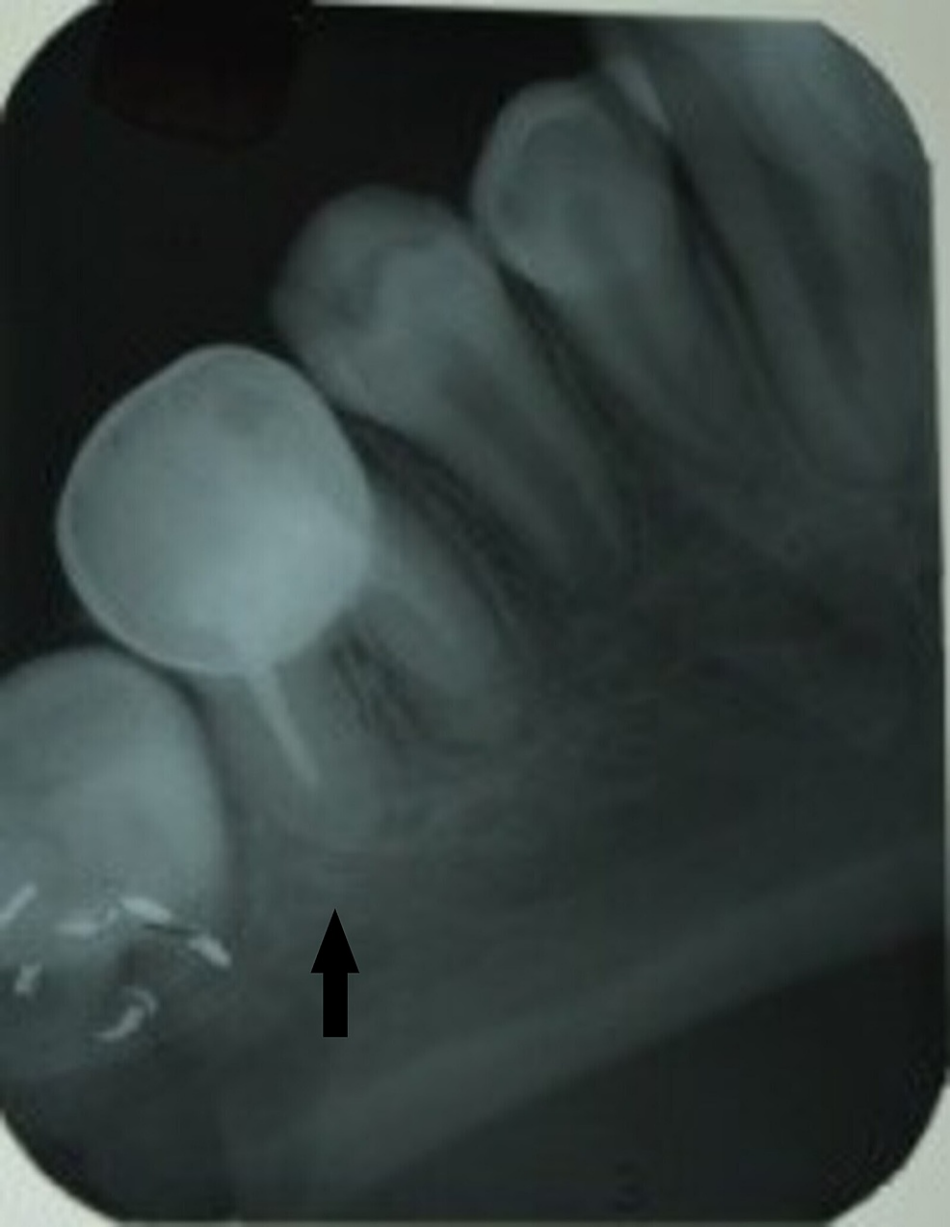
One-year follow-up - IOPA radiograph IOPA: intraoral periapical

**Figure 16:**
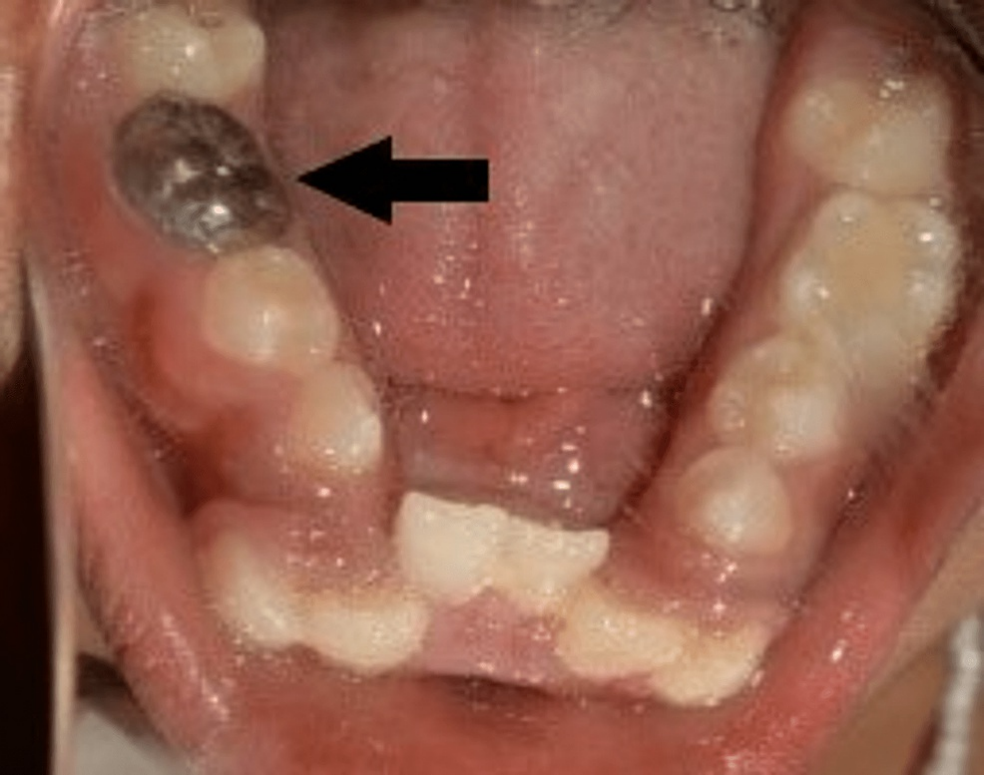
One-year follow-up image

The patient's uncooperative behaviour and a small jaw with decreased mouth opening were a challenge while treating the patient. With proper behaviour modification techniques and modification of instruments, treatment was completed over multiple visits. Endodontic files were bent to file the root canals. At the one-year follow-up, the patient was asymptomatic both clinically and radiographically (Figures 15, 16). The patient was kept under follow-up.

## Discussion

Genetic diseases have been on the rise, and this generally alarms dentists in terms of properly diagnosing and dealing with these patients. Prenatal three-dimensional sonography, MRI, and foteoscopy at mid-trimester can help diagnose Apert syndrome. The discovery of mutations in the FGFR gene enables definitive antenatal diagnosis. But in most cases, the diagnosis is confirmed at birth or during early infancy based on a thorough clinical evaluation and specialised tests like the molecular genetic test [15].

Treatment should start soon after birth with a proper diagnosis. Since it affects various systems, a multidisciplinary (respiratory, cerebral, dental, ophthalmic, and orthopaedic) approach is essential. Both surgical and supportive treatment are needed. An early craniotomy is required to prevent premature closure of sutures and complications such as elevated pressure. For functional and aesthetic improvements, surgical separation of digits, cosmetic reconstruction of the face can be undertaken. Early optimisation of hearing with possible hearing aids, airway management, psychological counselling, speech correction, and genetic counselling are required for comprehensive rehabilitation of the child in society. In patients with Apert syndrome, severe skeletal class III open bite malocclusion can be corrected by orthodontic treatment and orthognathic surgery. Conventional surgical advancement (Le Fort III osteotomy) of the midface or gradual advancement with osteogenic distraction (Ilizarov procedure) can be done later [16]. Oral hygiene maintenance is difficult in these patients because of hand deformities, malocclusion, and pseudo-cleft in the palate. Custom-made toothbrushes or the new generation of electric toothbrushes and fluoride mouth rinses may make the task easier. Professional care, including frequent dental examinations, oral hygiene prophylaxis, fluoride treatments, and dental sealants, will help to prevent dental diseases.

## Conclusions

Craniosynostosis is a major medical problem that is associated with considerable morbidity. Early diagnosis and surgical intervention are crucial for favorable outcomes. Advances in the medical field have increased the life span of patients with Apert syndrome. With an emphasis on parent and patient education, as well as preventive care and early detection and treatment of dental diseases, a dentist can help these patients to lead better lives.
